# Tooth loss and risk of cardiovascular disease and stroke: A dose-response meta analysis of prospective cohort studies

**DOI:** 10.1371/journal.pone.0194563

**Published:** 2018-03-28

**Authors:** Fei Cheng, Mi Zhang, Quan Wang, Haijun Xu, Xiao Dong, Zhen Gao, Jiajuan Chen, Yunjie Wei, Fen Qin

**Affiliations:** 1 Department of Cardiology, Taihe Hospital, Hubei University of Medicine, Shiyan, Hubei, China; 2 Department of Stomatology, Taihe Hospital, Hubei University of Medicine, Shiyan, Hubei, China; 3 Department of Obstetrics and Gynecology, Taihe Hospital, Hubei University of Medicine, Shiyan, Hubei, China; Kaohsiung Medical University Hospital, TAIWAN

## Abstract

Conflicting results identifying the association between tooth loss and cardiovascular disease and stroke have been reported. Therefore, a dose-response meta-analysis was performed to clarify and quantitatively assess the correlation between tooth loss and cardiovascular disease and stroke risk. Up to March 2017, seventeen cohort studies were included in current meta-analysis, involving a total of 879084 participants with 43750 incident cases. Our results showed statistically significant increment association between tooth loss and cardiovascular disease and stroke risk. Subgroups analysis indicated that tooth loss was associated with a significant risk of cardiovascular disease and stroke in Asia and Caucasian. Furthermore, tooth loss was associated with a significant risk of cardiovascular disease and stroke in fatal cases and nonfatal cases. Additionally, a significant dose-response relationship was observed between tooth loss and cardiovascular disease and stroke risk. Increasing per 2 of tooth loss was associated with a 3% increment of coronary heart disease risk; increasing per 2 of tooth loss was associated with a 3% increment of stroke risk. Subgroup meta-analyses in study design, study quality, number of participants and number of cases showed consistent findings. No publication bias was observed in this meta-analysis. Considering these promising results, tooth loss might provide harmful health benefits.

## Introduction

Cardiovascular disease affects millions of people in developed and developing countries that is now a public health crisis. Despite the decline in the mortality rate of developed countries, cardiovascular disease is still the main cause of death and has caused serious social and economic distress on a global scale over the past few decades. In low and middle-income countries, the incidence of cardiovascular disease has risen sharply [1–3]. By 2020, cardiovascular disease is expected to be the leading cause of morbidity and mortality in most developing countries[4]. The etiology of cardiovascular disease involves both genetic and environmental factors. Therefore, understanding the impact of environmental factors on cardiovascular disease will help to prevent cardiovascular disease.

Oral cavity is an important part of the body, and is starts in digestive system, mainly by the lip and cheek, tongue and palate, salivary glands, teeth and jaw, with mastication, swallowing, speech and feeling, and other functions, which maintain the normal shape of maxillofacial. Oral health is an important part of human health. The World Health Organization (WHO) identifies dental health as one of the top ten criteria for human health. Poor oral health may increase systemic inflammation, resulting in a local overly aggressive immune response, and thus could have important implications for cardiovascular disease. Periodontal disease and tooth loss are two common oral health measures[5]. Tooth loss has been considered to impact quality of life[6], and been known to considerably influence food choice, diet, nutrition intake, and esthetics[7].

Previous studies have examined the correlation between tooth loss and cardiovascular disease and stroke risk[8–24]. However, the result remains controversial. Additionally, no study to clarify and quantitative assessed tooth loss in relation to tooth loss and cardiovascular disease and stroke risk. Thus, we performed this dose-response meta-analysis to clarify and quantitative assessed the correlation between tooth loss and tooth loss and cardiovascular disease and stroke risk.

## Methods

Our meta-analysis was designed following the preferred reporting items for systematic reviews and meta-analyses (PRISMA Compliant) statement(S1 Checklist)[25].

### Search strategy

PubMed and EMBASE were searched for studies that contained risk estimates for the outcomes of coronary heart disease and were published update to April 2017, with keywords including “Coronary heart disease” [MeSH] OR”stroke” [MeSH] OR “Cardiovascular Diseases” [MeSH] OR “Coronary Disease” [MeSH] OR “myocardial infarction” [MeSH] AND “dentition” [MeSH] OR”tooth loss” [MeSH] OR”edentulous” [MeSH]. The search strategy is shown in detail in S1 List.

### Study selection

Two independent researchers investigate information the correlation between tooth loss and cardiovascular disease and stroke risk: outcome was coronary heart disease and stroke. To ensure the correct identification of qualified research, the two researchers read the reports independently, and the disagreements were resolved through consensus by all of the authors.

### Data extraction

Use standardized data collection tables to extract data. Each eligible article information was extracted by two independent researchers. We extracted the following information: first author; publication year; mean value of age; country; study name; sex; cases and participants; the categories of tooth loss; relative risk or odds ratio (OR). We collect the risk estimates with multivariable-adjusted[26]. According to the Newcastle-Ottawa scale, quality assessment was performed for non-randomized studies[27]. The disagreements were resolved through consensus by all of the authors.

### Statistical analysis

We pooled relative risk estimates to measure the association between tooth loss and cardiovascular disease and stroke risk; the hazard ratio were considered equivalent to the relative risk[28]. Results in different subgroups of tooth loss and cardiovascular disease and stroke risk were treated as two separate reports.

Due to different definitions cut-off points in the included studies for categories, we performed a relative risk estimates by using the method recommended by Greenland, Longnecker and Orsini and colleagues[29]. A flexible meta-regression based on restricted cubic spline (RCS) function was used to fit the potential non-linear trend, and generalized least-square method was used to estimate the parameters. This procedure treats tooth loss (continuous data) as an independent variable and logRR of diseases as a dependent variable, with both tails of the curve restricted to linear. A P value is calculated for linear or non-linear by testing the null hypothesis that the coefficient of the second spline is equal to zero[29].

We use STATA software 12.0 (STATA Corp, College Station, TX, USA) to evaluate the relationships between tooth loss and risk of coronary heart disease and stroke. By using Q test and I^2^ statistic to assess heterogeneity among studies. Random-effect model was chosen if P_Q_< 0.10 or I^2^>50%, otherwise, fixed-effect mode was applied. Begg’s and Egger’s tests were to assess the publication bias of each study. P< 0.05 was considered signifcant for all tests.

## Results

**Fig 1:**
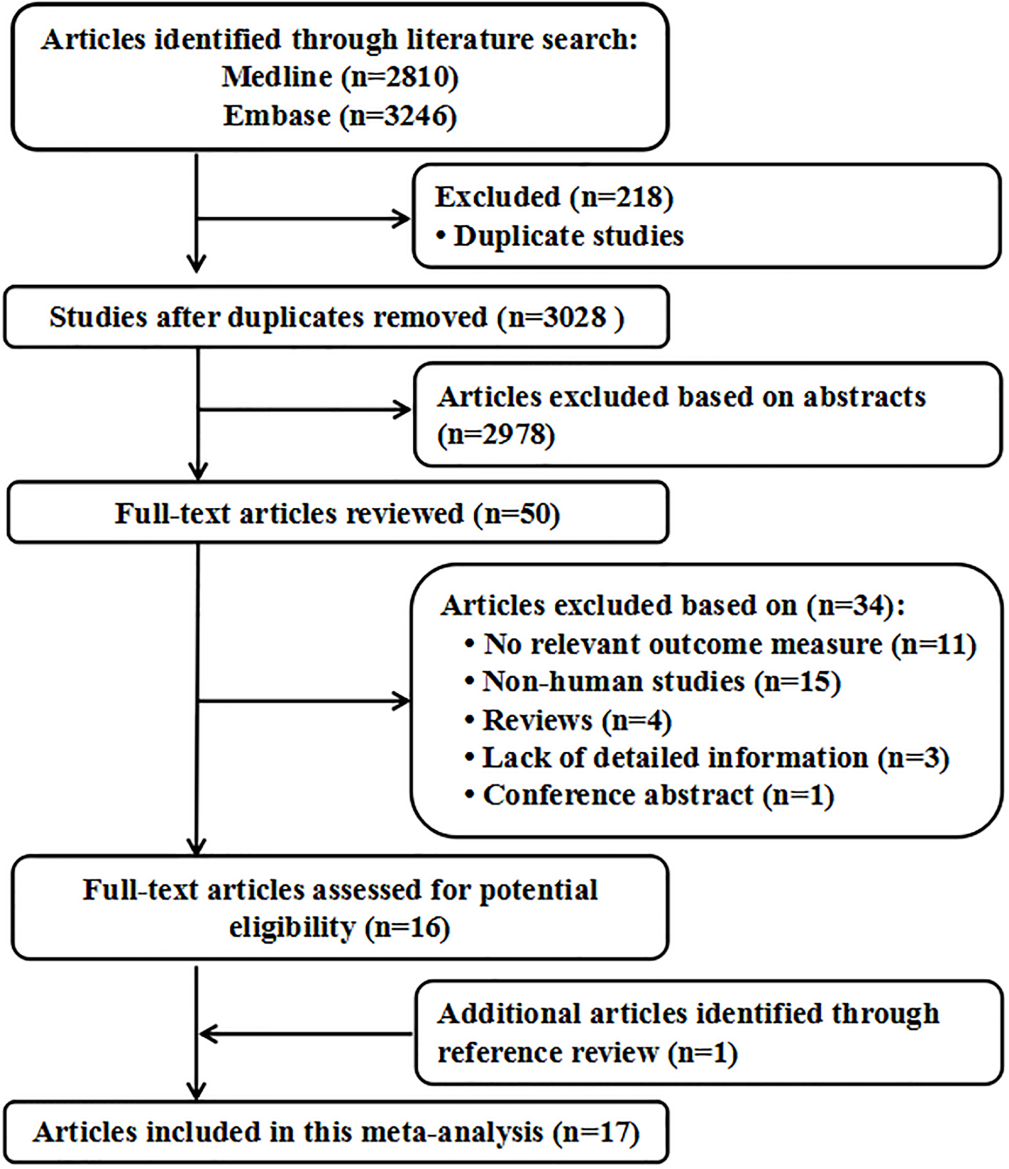
Flow diagram of the study selection process.

A total of 2810 studies from Medline, 3246 studies from Embase. After removing duplicates study, 3028 studies were identifed. reviewing their titles and abstracts, 2978 citations were excluded. The remaining 50 citations were assessed in more detail for eligibility by reading the full text. Among them, 11 studies were excluded due to no relevant outcome measure; 15 studies were excluded due to no human studies; 3 study was excluded due to lack of detailed information; 4 study was excluded due to review; 1 study was excluded due to conference abstract. After review reference of studies, one article was identified. Finally, 28 studies were used for the final data synthesis. The flow chart of literature searching was presented in Fig 1.

### Study characteristics

The characteristics of the included studies of tooth loss and risk of coronary heart disease and stroke are shown in the Tables 1 and 2.

**Table 1:** Characteristics of participants in included studies of tooth loss in relation to risk of coronary heart disease and stroke.

Author(year)	Study design	Country	Sex of population	Age at baseline (years)	No of participants	Endpoints (cases)	Quality score
Dietrich et al(2008)	Cohort	USA	Male	21–84	1203	Coronary Heart Disease(364)	8
Howell et al(2001)	Cohort	USA	Male	40–84	22037	Cardiovascular Disease(797) Stroke(631)	8
Hung et al(2004)	Cohort	USA	Male and Female	40–75	41407 men 58974women	Men Coronary Heart Disease(1656) Women Coronary Heart Disease(544)	8
Tuominen et al(2003)	Cohort	Finland	Male and Female	30–69	2518 men 2392 women	Men Coronary Heart Disease(566) Women Coronary Heart Disease(701)	8
Abnet et al(2005)	Cohort	China	Mix	40–69	29584	Cardiovascular Disease(1932) Stroke(2866)	8
Elter et al(2004)	Cohort	USA	Male	52–75	8363	Cardiovascular Disease(1619)	8
Holmlund et al(2010)	Cohort	Sweden	Male and Female	20–89	7674	Cardiovascular disease(299) Coronary heart disease(167) Stroke(83)	7
Hujoel et al(2000)	Cohort	USA	Mix	25–74	8032	Cardiovascular disease(1265)	8
Joshipura et al(1996)	Cohort	USA	Male	40–75	44119	Cardiovascular disease(757)	8
Joshy et al(2016)	Cohort	Australia	Mix	45–75	172630	Ischaemic heart disease(3239) Heart failure(212) Stroke(283)	7
Jung et al(2016)	Cohort	Korea	Mix	≥50	5359	Cardiovascular disease(536)	7
Liljestrand et al(2015)	Cohort	Finnish	Mix	25–74	8446	Cardiovascular disease(692) Coronary heart disease(482) Acutemyo cardial infarction(253) Stroke(268)	8
Noguchi et al(2015)	Cohort	Japan	Male	36–59	3081	Myocardial infarction	8
Schwahn et al(2013)	Cohort	Caucasian	Mix	64.0	1803	Cardiovascular disease(128)	7
Tu et al(2007)	Cohort	United Kingdom	Mix	≤30	12223	Cardiovascular disease(319) Coronary heart disease(222) Stroke(64)	8
Vedin et al(2015)	Cohort	Sweden	Mix	≥60	15456	Cardiovascular disease(705) Myocardial infarction(746) Stroke(301)	8
Watt et al(2012)	Cohort	Scotland	Mix	≥40	12871	Cardiovascular disease(297) Stroke(92)	8

**Table 2:** Outcomes and covariates of included studies of tooth loss in relation to risk of coronary heart disease and stroke.

Author(year)	Endpoints (cases)	Data source	Category and relative risk (95% CI)	Covariates in fully adjusted model
Dietrich et al(2008)	Coronary Heart Disease(364)	self-reports	Age <60 5 teeth lost, 1.0 (reference);7, 1.68 (1.13, 2.52); 10, 1.55 (0.94, 2.56);13, 2.12 (1.26, 3.60);Edentulous,1.90(0.92, 3.93) Age >60 6 teeth lost, 1.0 (reference);8, 0.90 (0.60, 1.35); 11, 1.13 (0.75, 1.69);17, 1.13(0.71, 1.78), Edentulous,1.95(1.18, 3.22)	Adjusted for age, body mass index, high-density lipoprotein cholesterol, total cholesterol, triglycerides, hypertension, mean systolic and diastolic blood pressure, diabetes mellitus, fasting glucose, smoking, alcohol intake, occupation and education, income, and marital status.
Hung et al(2004)	Coronary Heart Disease(1654)	self-reports	Men Nonfatal CHD <7 teeth lost, 1.0 (reference); >7-<15, 1.10 (0.95, 1.26);>16-<21, 1.35 (1.06, 1.72); >22, 1.36(1.11, 1.67) Fatal CHD <7 teeth lost, 1.0 (reference); >7-<15, 1.26 (1.01, 1.57);>16-<21, 1.19 (0.79, 1.80); >22, 1.79(1.34, 2.40) Women Nonfatal CHD <7 teeth lost, 1.0 (reference); >7-<15,1.14 (0.92, 1.42);>16-<21,1.34 (0.97, 1.87); >22, 1.64 (1.31, 2.05) Fatal CHD <7 teeth lost, 1.0 (reference); >7-<15, 1.02 (0.66, 1.55);>16-<21, 1.07 (0.56, 2.05); >22, 1.65 1.11,2.46)	Adjusted for age, smoking, alcohol consumption, body mass index, physical activity, family history of myocardialinfarction, multivitamin supplementuse, vitaminE use, history of hypertension, diabetes, and hypercholesterolemia in both cohorts and professionsfor men only, and for women only, menopausalstatusand hormoneuse
Joshipura et al(1996)	Cardiovascular disease(757)	self-reports	<7 teeth lost, 1.0 (reference); >7-<15, 1.03 (0.83, 1.27);>16-<21, 1.04 (0.71, 1.54); >22, 1.29(0.96, 1.73)	Adjusted for age; body mass index; exercise; smoking habits; alcohol consumption; family history of myocardial infarction before 60 years of age; vitamin E
Joshy et al(2016)	Ischaemic heart disease(3239) Heart failure(212) Stroke(283)	self-reports	Ischaemic heart disease <10 teeth lost, 1.0 (reference); >10-<22, 1.05 (0.96, 1.15);>22-<31, 1.20 (1.06, 1.35); 32, 1.10(0.95, 1.26) Heart failure <10 teeth lost, 1.0 (reference); >10-<22, 1.50 (1.04, 2.18);>22-<31, 2.04 (1.35, 3.09); 32, 1.97(1.27, 3.07) Stroke <10 teeth lost, 1.0 (reference); >10-<22, 1.11 (0.72, 1.73);>22-<31, 0.90 (0.59, 1.40); 32, 1.20(0.90, 1.62)	Adjusted for age; sex; tobacco smoking, alcohol consumption, Australian born status, region of residence, education, health insurance, physical activity and body mass index, with missing values in covariates were coded as a separate categories.
Schwahn et al(2013)	Cardiovascular disease(128)	self-reports	0 teeth lost, 1.0 (reference); >1-<9, 1.05 (0.67, 1.65);>10-<19, 1.08 (0.68, 1.71)	Adjusted for age, sex, education, marital status, partnership, smoking, risky alcohol consumption, physical activity, diagnosed diabetes mellitus, obesity, and hypertension.
Wiener et al(2014)	Stroke(17547)	self-reports	0 teeth lost, 1.0 (reference); >1-<5, 1.29 (1.17, 1.42);>6-<32, 1.68 (1.50, 1.88); 32, 1.86(1.63, 2.11)	Adjusted for sex, race/ethnicity, age, education, income levelm, health insurance, smoking status, physical activity outside of work, dental visits within the previous year, heavy drinking, diabetes and BMI.

### Tooth loss and cardiovascular disease risk

**Fig 2:**
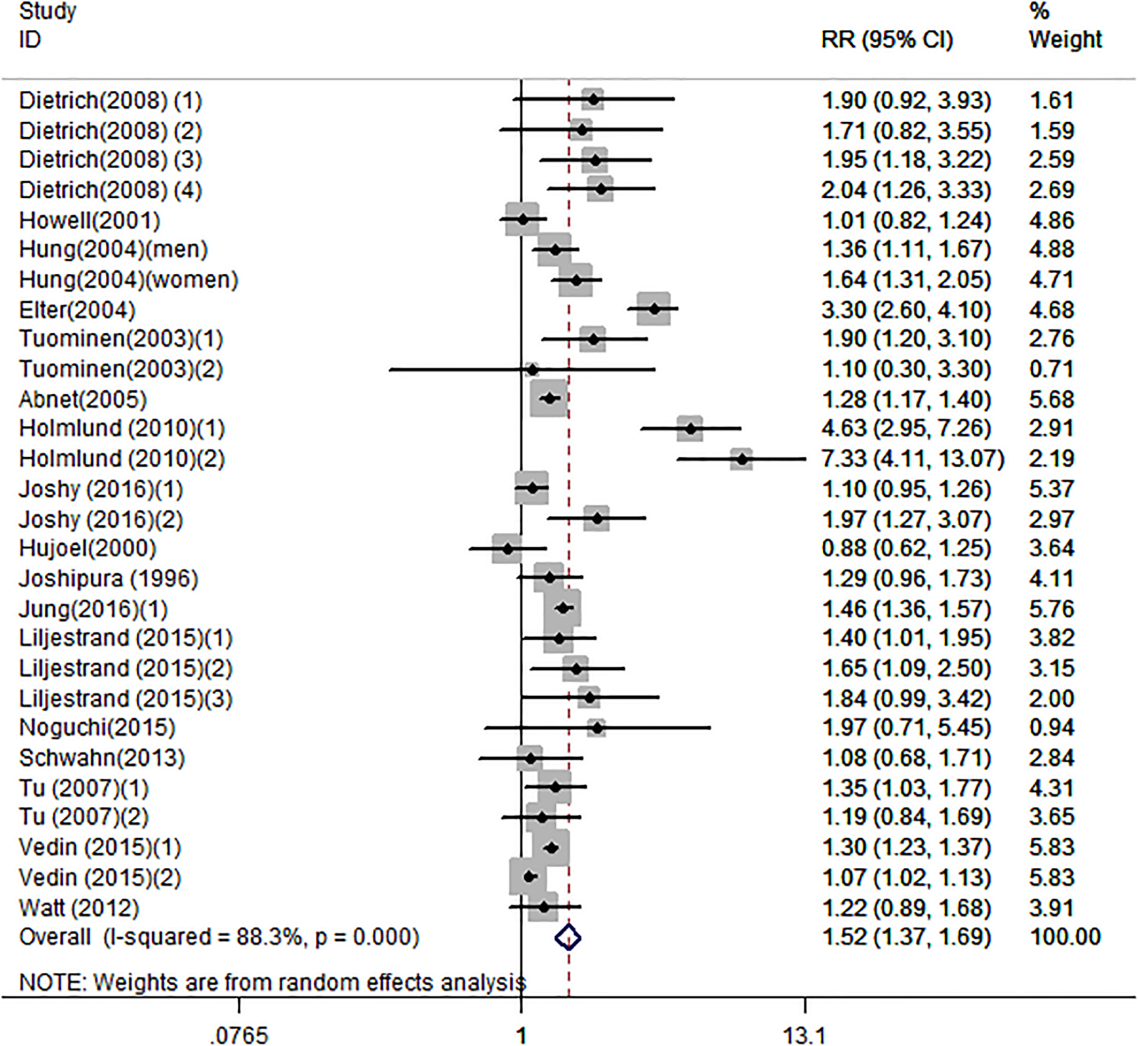
Forest plots for meta-analysis of tooth loss and risk of cardiovascular disease.

**Fig 3:**
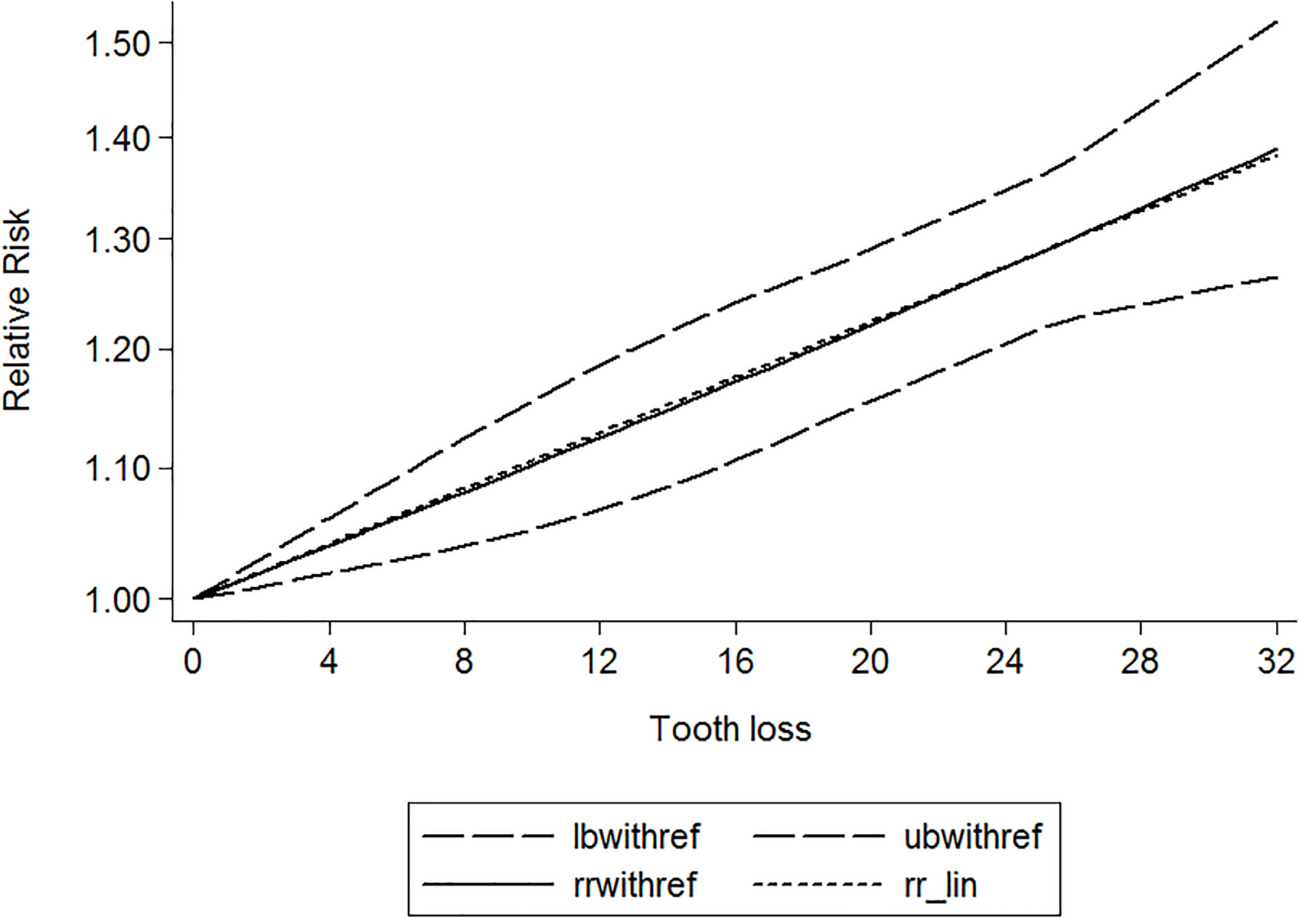
Dose-response relationship between tooth loss and risk of cardiovascular disease.

Twenty-eight independent reports from seventeen studies investigated the association between tooth loss and risk of coronary heart disease. Compared with the lowest tooth loss, tooth loss is significantly associated with a higher risk of cardiovascular disease (RR:1.52; 95% CI, 1.37–1.69; *P* < .001)(Fig 2). Subgroups analysis indicated that tooth loss is associated with a significantly increasement of coronary heart disease risk in Asia (RR:1.38; 95% CI, 1.21–1.56; *P* < .001)(Table 3) and Caucasian(RR:1.55; 95% CI, 1.35–1.75; *P* < .001)(Table 3). Furthermore, tooth loss was associated with a significantly cardiovascular disease risk in fatal cardiovascular disease (RR:1.31; 95% CI, 1.19–1.43; *P* < .001) (Table 3) and nonfatal cardiovascular disease (RR:1.56; 95% CI, 1.27–1.85; *P* < .001) (Table 3). Also, tooth loss was associated with a significantly risk of coronary heart disease in male (RR:1.92; 95% CI, 1.34–2.50; *P* < .001) (Table 3) and female (RR:1.48; 95% CI, 1.20–1.76; *P* < .001) (Table 3). Additionally, a significant dose-response relationship was observed between tooth loss and coronary heart disease risk. Increasing per 2 of tooth loss was associated with a 3% increment of coronary heart disease risk (RR:1.03; 95% CI, 1.02–1.04; *P* < .001) (Fig 3).

**Table 3:** Stratified analyses of relative risk of coronary heart disease and stroke.

	No of reports	Relative risk (95% CI)	P for heterogeneity	I^2^	P for test
**Coronary heart disease**					
Total cases	28	1.52(1.37–1.69)	0.000	88.3%	<0.001
Fatal cases	13	1.31(1.19–1.43)	0.000	69.7%	<0.001
Nonfatal cases	15	1.56(1.27–1.85)	0.000	89.1%	<0.001
Subgroup analysis for total coronary heart disease					
Sex					
Male	6	1.92(1.34–2.50)	0.000	78.6%	<0.001
Female	6	1.48(1.20–1.76)	0.137	40.2	<0.001
Study location					
Caucasia	25	1.55(1.35–1.75)	0.000	85.7%	<0.001
Asia	3	1.38(1.21–1.56)	0.068	62.8%	<0.001
No of participants					
≥10 000	10	1.33(1.26–1.40)	0.060	54.9%	<0.001
<10 000	18	1.51(1.35–1.67)	0.000	80.0%	<0.001
No of cases					
≥500	14	1.50(1.36–1.65)	0.000	81.8%	<0.001
<500	14	1.43(1.23–1.62)	0.000	77.7%	<0.001
**Stroke**					
**Subgroup analyses for total stroke**					
Total cases	8	1.18(1.11–1.25)	0.000	46.7%	<0.001
Study location					
Caucasia	5	1.25(1.18–1.32)	0.001	67.8%	<0.001
Asia	3	1.12(1.01–1.23)	0.528	0.0%	<0.001
No of participants					
≥10 000	4	1.43(1.27–1.60)	0.000	88.9%	<0.001
<10 000	4	1.08(1.02–1.15)	0.643	0.0%	<0.001
No of cases					
≥500	6	1.30(1.17–1.44)	0.000	74.9%	<0.001
<500	2	1.09(1.03–1.15)	0.590	0.0%	<0.001

### Tooth loss and stroke risk

**Fig 4:**
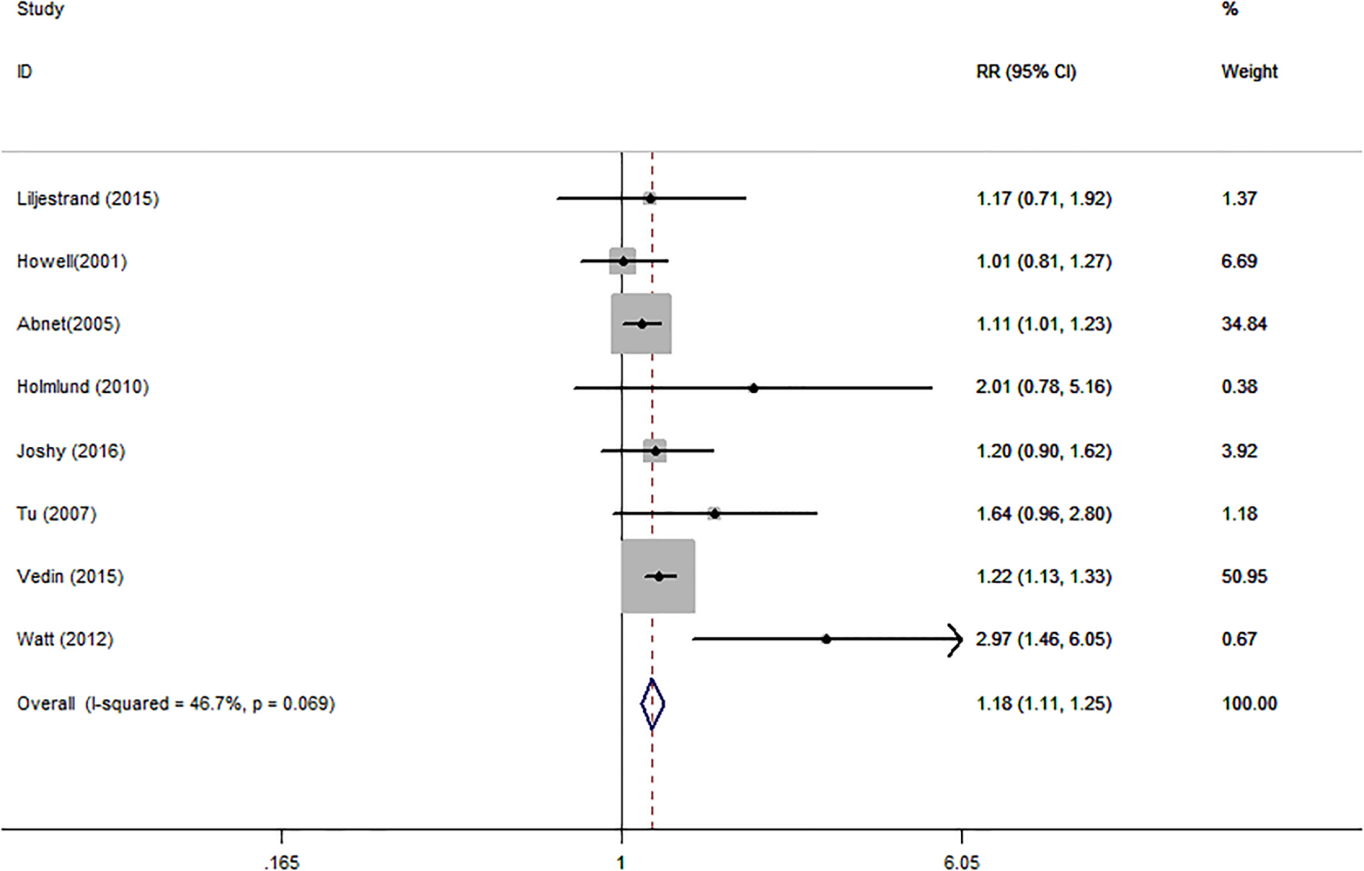
Forest plots for meta-analysis of tooth loss and risk of stroke.

**Fig 5:**
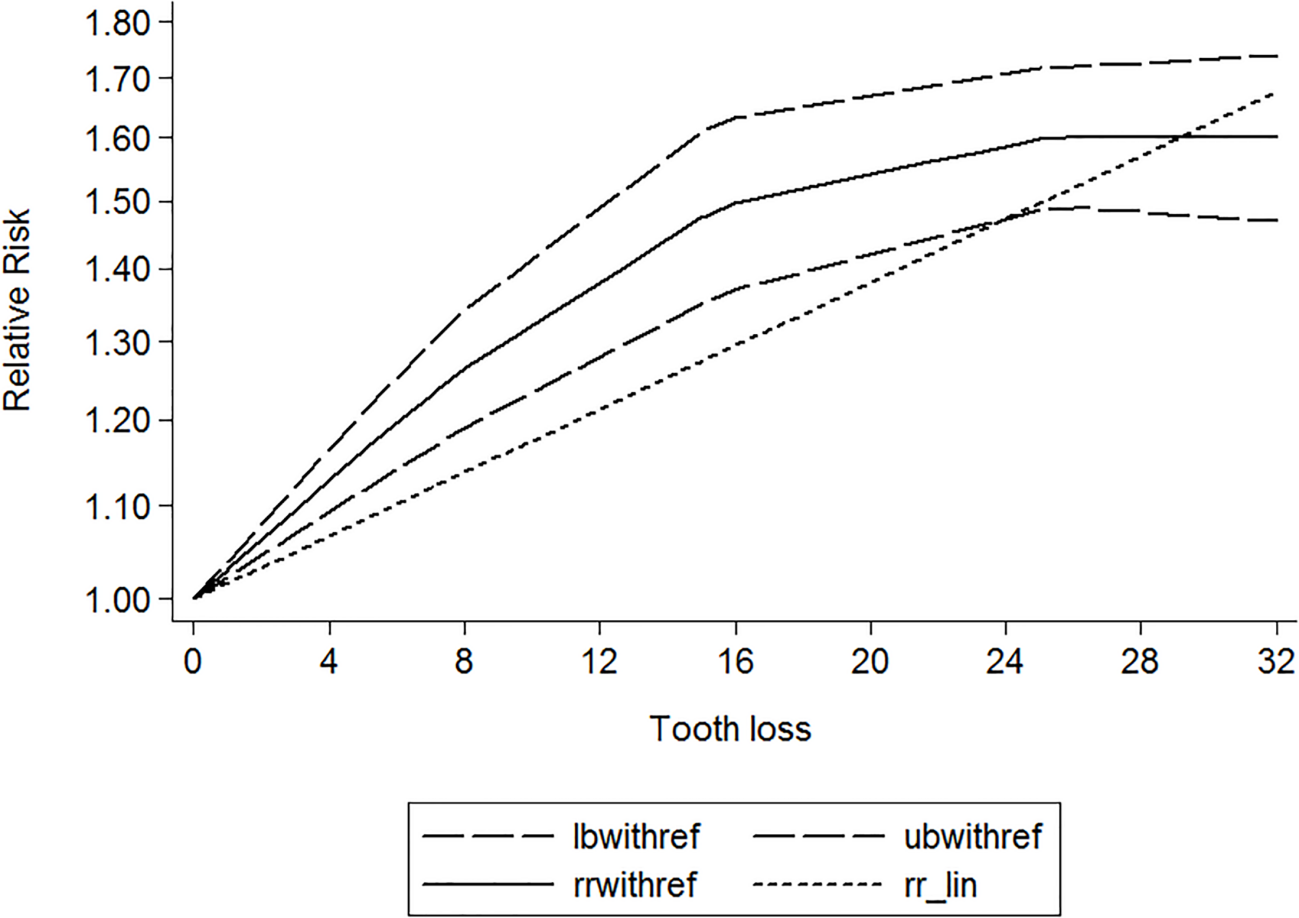
Dose-response relationship between tooth loss and risk of stroke.

Eight independent reports from eight studies investigated the association between tooth loss and risk of stroke. Compared with the lowest tooth loss, tooth loss is significantly associated with a higher risk of stroke (RR:1.18; 95% CI, 1.11–1.25; *P* < .001)(Fig 4). Subgroups analysis indicated that tooth loss is associated with a significantly incensement of stroke risk in Asia (RR:1.12; 95% CI, 1.01–1.23; *P* < .001) (Table 3) and Caucasian (RR:1.25; 95% CI, 1.18–1.32; *P* < .001) (Table 3). Additionally, a significant dose-response relationship was observed between tooth loss and stroke risk. Increasing per 2 of tooth loss was associated with a 3% increment of stroke risk (RR:1.03; 95% CI, 1.02–1.04; *P* < .001) (Fig 5).

### Subgroup analyses

Subgroup analysis was performed to check the stability of the primary outcome. Subgroup meta-analyses in study design, study quality, number of participants and number of cases showed consistent findings (Table 3).

### Publication bias

Each studies in this meta-analysis were performed to evaluate the publication bias by both Begg’s funnel plot and Egger’s test. P>0.05 was considered no publication bias. The results show no obvious evidence of publication bias was found in the relationship between tooth loss and cardiovascular disease and stroke risk (S1 Table). A funnel plot for publication bias assessment is illustrated in S1 and S2 Figs.

## Discussion

Recently, tooth loss has been found to be associated with decreased risks of lung cancer, colorectal cancer, prostate cancer, breast cancer and pancreatic cancer[30]. However, as for tooth loss and coronary heart disease and stroke risk, there are several unsolved issues. First, the relationship between tooth loss and cardiovascular disease and stroke risk is remains controversial. Some studies found that tooth loss was associated with a increase risk of coronary heart disease and stroke, whereas others failed to fnd relationship between tooth loss and cardiovascular disease and stroke risk. Furthermore, the dose-response relationship between tooth loss and coronary heart disease and stroke risk has not been described.

In the current meta-analysis was based on seventeen cohort study, with a total of 879084 participants with 43750 incident cases. Thus, this meta analysis provides the most up-to-date epidemiological evidence supporting tooth loss is harmful for cardiovascular disease and stroke. A dose-response analysis revealed that increasing tooth loss (per 2 increment) was associated with a 3% increment of coronary heart disease risk, increasing tooth loss (per 2 increment) was associated with a 3% increment of stroke risk. Furthermore, tooth loss was associated with a significantly cardiovascular disease and stroke risk in Asia and Caucasian. Also, tooth loss was associated with a significantly risk of cardiovascular disease in male and female. Additionally, tooth loss was associated with a significantly cardiovascular disease and stroke risk in fatal cases and nonfatal cases. Subgroup meta-analyses by various factors also showed consistent findings.

Several plausible pathways may reasonable for the relationship between tooth loss and cancer. The influence of chronic inflammation on cancer development is one possible pathwa. Chronic systemic inflammation linked to periodontal disease. People with few or no teeth would this have an increased risk of systemic diseases such as cardiovascular disease and stroke[31]. Secondly, the main cause of teeth loss is dental caries, and carbohydrate intake is the dental caries cause. Carbohydrate intake was associated with increased risk cardiovascular disease and stroke, therefore teeth loss indirect effects risk of cardiovascular disease and stroke[32]. Third, the progress of tooth damage destroys normal periodontal tissue, allowing oral microbial accumulation deep into oral tissue, thereby promoting its growth, thus resulting in cardiovascular disease and stroke[33]. Fourth, Tooth loss is the ultimate stage of periodontal disease and may be associated with an increase in C-reactive protein (CRP), which itself is implicated in atherosclerosis and thus in the occurrence of stroke[34]. Thus, tooth loss and cancer seems to be closely related.

To our knowledge, this is the first study to identify and quantify the potential dose-response association between tooth loss and cardiovascular disease and stroke risk in a large cohort of both men and women. Although, we performed this meta-analysis very carefully, however, some limitations must be considered in the current meta-analysis. First, we only select literature that written by English, which may have resulted in a language or cultural bias, other language should be chosen in the further. Second, in the subgroup analysis in different ethnic population, there might be insufficient statistical power to check an association.

In conclusion, our dose–response meta-analysis suggests tooth loss was independently associated with deleterious coronary heart disease and stroke risk increment. In the future, large-scale and population based association studies must be performed to help identify the putative causal role that tooth loss plays in increasing the incidence of these diseases.

## Supporting information

S1 Table Publication bias analysis of the meta-analysis. (DOCX)

S1 Fig Begg’s funnel plot for assessment of publication bias of tooth loss and risk of cardiovascular disease. (TIF)

S2 Fig Begg’s funnel plot for assessment of publication bias of tooth loss and risk of stroke. (TIF)

S1 List The detail of search strategy. (DOCX)

S1 Checklist The detail of PRISMA checklist. (DOC)
