# Supplementary material for: Tooth loss and risk of cardiovascular disease and stroke: A dose-response meta analysis of prospective cohort studies
Source: PLoS One. 2018 Mar 28;13(3):e0194563. doi: 10.1371/journal.pone.0194563 (PMC5874035; doi:10.1371/journal.pone.0194563)
Supplement: S1 Table — (DOCX) [file pone.0194563.s001.docx]

**Supplementary table 1. Publication bias analysis of the meta-analysis**

|  | Test | t | 95% CI | P |
| --- | --- | --- | --- | --- |
| **Coronary heart disease** | Begg’s test |  |  | 0.311 |
|  | Egger’s test | 2.6 | -0.4,4.9 | 0.126 |
| **Stroke** | Begg’s test |  |  | 0.646 |
|  | Egger’s test | 0.25 | -0.77,2.72 | 0.246 |
