# Supplementary figures and images for: Tooth loss and risk of cardiovascular disease and stroke: A dose-response meta analysis of prospective cohort studies
Source: PLoS One. 2018 Mar 28;13(3):e0194563. doi: 10.1371/journal.pone.0194563 (PMC5874035; doi:10.1371/journal.pone.0194563)

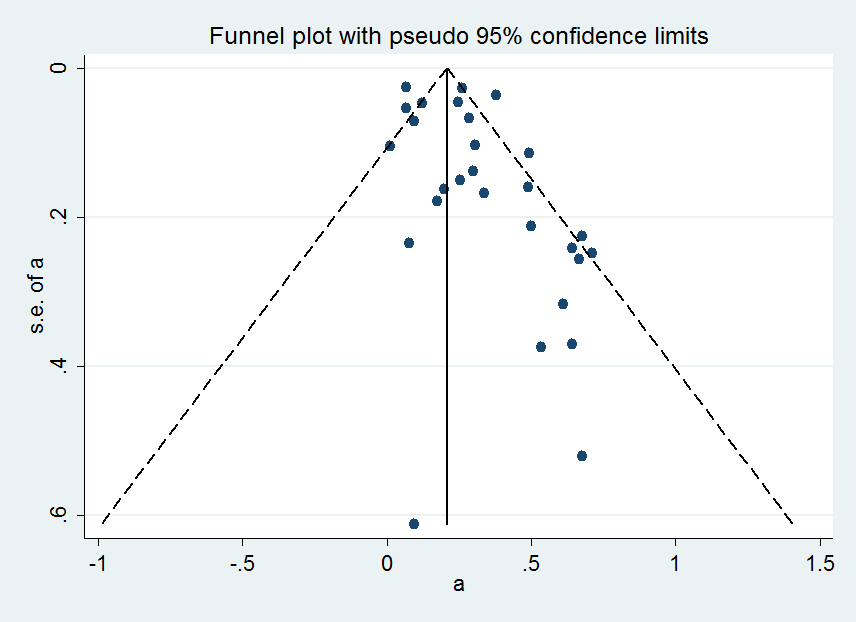

Supplement: S1 Fig — (TIF) [file pone.0194563.s002.tif]

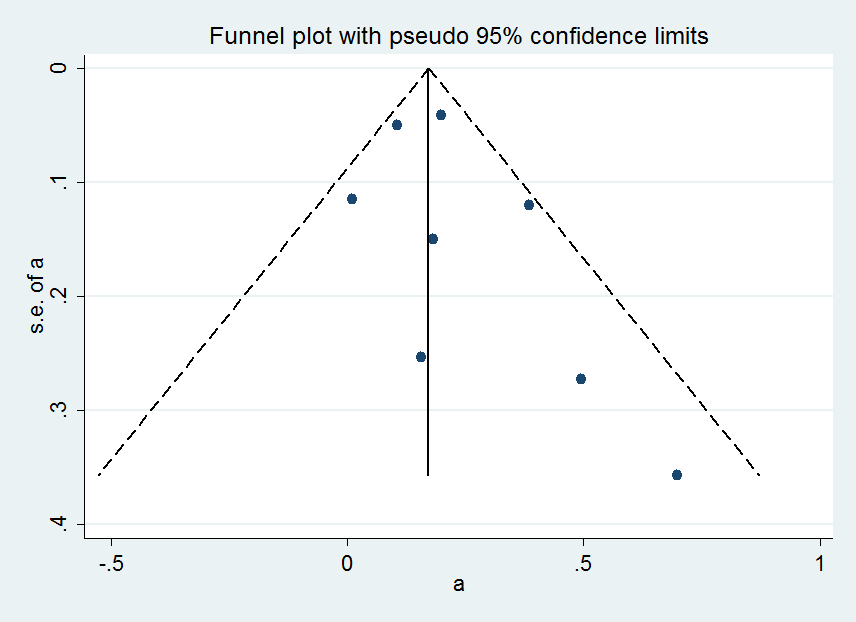

Supplement: S2 Fig — (TIF) [file pone.0194563.s003.tif]
