# Supplementary material for: Tooth loss and risk of cardiovascular disease and stroke: A dose-response meta analysis of prospective cohort studies
Source: PLoS One. 2018 Mar 28;13(3):e0194563. doi: 10.1371/journal.pone.0194563 (PMC5874035; doi:10.1371/journal.pone.0194563)
Supplement: S1 List — (DOCX) [file pone.0194563.s004.docx]

**Supplementary List 1 Search Strategy**

**PubMed**

#1 (((Coronary heart disease*[Title/Abstract]) OR stroke*[Title/Abstract]) OR Cardiovascular Diseases*[Title/Abstract]) OR Coronary Disease*[Title/Abstract] OR myocardial infarction*[Title/Abstract]

#2 (((dentition*[Title/Abstract]) OR "tooth loss*"[Title/Abstract])

#3 #1 AND #2

**EMBASE**

#1 dentition* AND ([article]/lim OR [article in press]/lim OR [conference abstract]/lim OR [conference paper]/lim) AND ([chinese]/lim OR

[english]/lim) AND [embase]/lim

#2 tooth loss* AND ([article]/lim OR [article in press]/lim OR [conference abstract]/lim OR [conference paper]/lim) AND ([chinese]/lim OR [english]/lim) AND [embase]/lim

#12 #1 OR #2

#13 Coronary heart disease* AND ([article]/lim OR [article in press]/lim OR [conference abstract]/lim OR [conference paper]/lim) AND ([chinese]/lim OR

[english]/lim) AND [embase]/lim

#14 stroke* AND ([article]/lim OR [article in press]/lim OR [conference abstract]/lim OR [conference paper]/lim) AND ([chinese]/lim

OR [english]/lim) AND [embase]/lim

#15 Cardiovascular Diseases* AND ([article]/lim OR [article in press]/lim OR [conference abstract]/lim OR [conference paper]/lim) AND ([chinese]/lim

OR [english]/lim) AND [embase]/lim

#16 Coronary Disease* AND ([article]/lim OR [article in press]/lim OR [conference abstract]/lim OR [conference paper]/lim) AND ([chinese]/lim

OR [english]/lim) AND [embase]/lim

#17 #13 OR #14 OR #15 OR #16

#18 #12 AND #17
